# Evaluation of MC3T3 Cells Proliferation and Drug Release Study from Sodium Hyaluronate-1,4-butanediol Diglycidyl Ether Patterned Gel

**DOI:** 10.3390/nano7100328

**Published:** 2017-10-14

**Authors:** Sumi Bang, Dipankar Das, Jiyun Yu, Insup Noh

**Affiliations:** 1 Convergence Institute of Biomedical Engineering and Biomaterials, Seoul National University of Science of Technology, Seoul 01811, Korea; bobosumi48@gmail.com (B.S.); dipankardas@seoultech.ac.kr (D.D.); 2 Department of Chemical and Biomolecular Engineering, Seoul National University of Science of Technology, 232 Gongneung-ro, Nowon-gu, Seoul 01811, Korea; jiyun_@seoultech.ac.kr

**Keywords:** cell proliferation, bone regeneration, hyaluronate, MC3T3 cell, pattern gel

## Abstract

A pattern gel has been fabricated using sodium hyaluronate (HA) and 1,4-butanediol diglycidyl ether (BDDGE) through the micro-molding technique. The cellular behavior of osteoblast cells (MC3T3) in the presence and absence of dimethyloxalylglycine (DMOG) and sodium borate (NaB) in the pattern gel (HA-BDDGE) has been evaluated for its potential application in bone regeneration. The Fourier transform infrared spectroscopy (FTIR), ^13^C-nuclear magnetic resonance spectroscopy (^13^C NMR), and thermogravimetric analysis (TGA) results implied the crosslinking reaction between HA and BDDGE. The scanning electron microscopy (SEM) analysis confirmed the formation of pattern on the surface of HA-BDDGE. The gel property of the crosslinked HA-BDDGE has been investigated by swelling study in distilled water at 37 °C. The HA-BDDGE gel releases DMOG in a controlled way for up to seven days in water at 37 °C. The synthesized gel is biocompatible and the bolus drug delivery results indicated that the DMOG containing patterned gel demonstrates a better cell migration ability on the surface than NaB. For local delivery, the pattern gel with 300 µM NaB or 300 µM DMOG induced cell clusters formation, and the gel with 150 µM NaB/DMOG showed high cell proliferation capability only. The vital role of NaB for bone regeneration has been endorsed from the formation of cell clusters in presence of NaB in the media. The in vitro results indicated that the pattern gel showed angiogenic and osteogenic responses with good ALP activity and enhanced HIF-1α, and Runx2 levels in the presence of DMOG and NaB in MC3T3 cells. Hence, the HA-BDDGE gel could be used in bone regeneration application.

## 1. Introduction

The development of size and shape specific biologically applicable hydrogels have opened inventive prospects in addressing challenges in tissue engineering like tissue architecture, vascularization, and cell seeding [1]. Hydrogels are water-swollen, physically or chemically crosslinked polymers which are remarkable in regenerative tissue engineering because of their capability to mimic the physical characteristics of tissues [2,3]. One of the precise advantages of hydrogels is their simple treating conditions, which allow directly for cell encapsulation in the gel [2]. The capacity to seed or encapsulate cells within a three-dimensional (3-D) network has distinct significance, because these substrates well replicate in vivo microenvironments than that of cell seeding on two-dimensional (2-D) materials [2,4,5].

For in vitro tissue engineering and scaffold designs, the precise spatial mechanism and association of cells are vital to characterize the appropriate microenvironment around the cells, simulating in vivo physical and chemical cues [6]. Cell–cell contact, biomolecules delivery, and tissue architecture are the main factors that regulate cell behaviors. Even though, in tissue engineered scaffolds, cells have self-assemble capacity to recover essential features of their cell-cell interactions, while, many of the interactions are eternally disappeared in the time of tissue isolation and seeding processes. Moreover, the homogeneous cell seeding throughout the scaffolds is quite difficult, because of the presence of a large number of cells in the border of scaffolds. The recent archetype of tissue engineering is concentrated on the application of cell-seeded scaffolds for in vitro generation of tissues and subsequent in vivo implantation [7]. Surface topography has been employed for stimulating the cellular orientation and monitoring the biological activity of cells within the structure of constructs [7]. However, the main drawbacks of in vitro tissue engineering is the failure to recapitulate cell dense paradigms with the required variety in cell types and structural complexity to mimic native tissues [8]. In vivo, cells are strongly organized in 3D architectures controlled by factors like cell-cell, cell-extracellular matrix (ECM), and cell-soluble factors [9]. Bioactive signals and stem cells that can respond to biomimetic morphogens and scaffolds are also important requisites in tissue engineering [10]. Bioactive signals demonstrate numerous vital roles on cell function and behavior. In most biological studies, soluble biochemical signals, for instance, growth factors or cytokines are added directly into the media to preserve and/or control cell behaviors in vitro. Conversely, these systems cannot precisely mimic convinced in vivo biological signaling motifs, which are regularly immobilized to ECM and also exhibit spatial gradients, which are critical for tissue morphology. In addition, biochemical cues and biophysical characteristics, for example, material hardness can influence cell activities but is not simple to control in conventional cell culturing practices [11]. One of the biggest challenges in tissue engineering is mass transfer limitations. This is the limiting factor in the size of any tissue developed in vitro, in addition to the successive incorporation of these constructs in vivo [12]. In vitro viable tissue-like systems frequently display dimensions beyond convenient perfusion limits, and have no functional blood vessels with flowing blood to supply nutrients and oxygen, and to remove waste products [13].

Currently, tissue engineering has turned into one of the most universally exploited approaches for cartilage and bone tissue reconstruction and regeneration [14]. The commonly used techniques for bone repair, like autografting and allografting are restricted due to the risks of donor-site morbidity, potential infection, and a high non-union rate with host tissues. Bone defects are one of the principal reasons of morbidity and disability in aged patients. Consequently, developing a technique to perfectly and enduringly repair the damaged cartilage and bone tissue is of noteworthy clinical attention for patients with cartilage lesions and bone defects. Preferably, for clinical application, the scaffolds of both cartilage and bone tissue engineering should be porous, highly biocompatible, nontoxic, and competent of endorsing cell differentiation and new tissue formation. They should also have stable mechanical properties, degrade in response to the formation of new tissue, facilitate the diffusion of nutrients and metabolites, adhere and integrate with the surrounding native tissue, and properly fill the injured site. Till date, a variety of biopolymers based hydrogels and hydrogels composites were employed in vitro and in vivo tissue engineering, especially for bone regeneration. For example, Han et al. developed alginate-based hybrid hydrogel that efficiently promotes the adhesion, proliferation, and differentiation of osteogenic and angiogenic cells [15]. Kook et al. prepared collagen-based sponge, which simulated natural bone tissue and supports cellular activity by enhancing cell adhesion and proliferation [16]. Vo et al. designed *N*-isopropylacrylamide/gelatin-based composite hydrogel, which enhanced bony bridging and mineralization within the defect and direct bone-implant contact [17]. Fu et al. designed PEG–PCL–PEG, collagen, and nanohydroxyapatite-based hydrogel that demonstrated a good biocompatibility and in vivo studies showed better performance in bone regeneration [18]. Dhivyaet et al. reported chitosan/nanohydroxyapatite/β-glycerophosphate-based hydrogel for in vivo studies in a rat bone defect, where the hydrogel accelerated bone formation at molecular and cellular levels [19]. Vishnu Priya et al. developed chitin and poly(butylene succinate) based hydrogel, which enhanced the initiation of differentiation and expression of alkaline phosphatase and osteocalcin, thus indicating its promise for regenerating irregular bone defects [20]. In vivo study of bone regeneration using alginate-bone ECM hydrogels was also reported by Gothard et al. [21]. A clinical study for bone generation was performed by Laino et al. [22]. They reported a comparative study of the histological aspects of bone formation in atrophic posterior mandibles augmented by autologous bone block from chin area with corticocancellous bone block allograft used as inlays with the sandwich technique [22]. Biomimetic mineralization on a macroporous cellulose-based matrix for bone regeneration was reported by Petrauskaite et al. [23] The porous cellulose matrix was non-cytotoxic, allowed the adhesion and proliferation of human osteoblastic cells, while both properties were improved on the mineralized cellulose matrices [23].

Our aim was to develop a shape-specific, hyaluronate-based patterned hydrogel for its application to bone regeneration. Sodium hyaluronate was chosen owing to its unique properties like abundant as natural ECM in human body, and its physico-chemical and immune-neutral characteristics [24,25]. The valuable properties led to development of various hyaluronic acid (HA) hydrogels for biomedical applications such as dermal fillers [26,27], cartilage regeneration [28,29], nucleus pulposus regeneration [30], and wound healing [31,32]. The microfabrication method was employed to pattern gel because of its noteworthy significance in tissue engineering as it can be employed to replicate structures (0.1–10 µm), to regulate the microenvironment of individual cells (10–400 µm), to control the structure of clusters of cells (>400 µm), and to control the interactions between multiple cell clusters [1]. In this aspect, soft lithography method has been established as an economical and effective process for patterning of bare glass [33] or metal-coated glass [34], polystyrene materials to flexible poly(dimethyl siloxane) (PDMS) materials [35], and biomaterials [36]. Soft lithography includes stamps fabricated from an elastomer or soft material like PDMS [6]. The PDMS stamp can mark ECM, self-assembled monolayer (SAM), and hydrogel to print on materials [6,24]. The well-defined ECM micro-patterns showed a significant effect on numerous imperative cell behaviors, such as cell adhesion and spreading [37], cell proliferation and differentiation [38], cell polarity [39], and migration [40]. 

In this study, a pattered hydrogel has been fabricated using sodium hyaluronate (HA) as biopolymer, 1,4-butanediol diglycidyl ether (BDDGE) as crosslinker, and sodium hydroxide (NaOH) as base by the micro-molding technique. BDDGE has been selected because of the presence of two epoxy rings, where, neucleophilile can attack on both ends and crosslinking will take place in HA. Scanning electron microscopy (SEM) analysis confirmed the formation of uniform pattern on the surface of gel. The hydrogel is biocompatible against MC3T3 cells. The HA-BDDGE gel releases dimethyloxalylglycine (DMOG) in a controlled manner for up to seven days in distilled water at 37 °C. It is observed that the hydrogel with more than 100 µM NaB showed MC3T3 cell clusters formation at day seven. In the cell proliferation study, the system with bolus drug delivery showed the best cell proliferations at the concentrations of 100 µM NaB and DMOG, individually. The presence of NaB helps the formation of MC3T3 cell clusters, supporting the vital role of NaB in bone regeneration. It has been also noticed that when drugs were delivered locally, the HA-BDDGE patterned gel showed higher intensity of ALP and Runx2, indicating a better bone regeneration ability. Although the experiment results demonstrated significant bone regeneration characteristics of the HA-BDDGE gel, still this study has some limitations that are associated with the in vitro study along with the lack of porosity in hydrogel and absence of adequate mechanical strength. Thus, to get better efficiency and the perfect ability for clinical and surgical experiments further modification would be appreciated in future. With the variation of amount of crosslinker and by mineralization with inorganic particles or ceramics, hydrogels scaffolds with different porosity and adequate mechanical property could be achieved. Furthermore, with the incorporation of biological matrix, such as gelation or collagen in the hydrogel network cell adhesiveness and tissue regeneration could be improved in future for use in a clinical study. Finally, the HA-BDDGE gel, with well micro-patterned architecture, biocompatibility, controlled release ability of DMOG drug, clusters formation ability of MC3T3 cells and higher intensity in alkaline phosphatase activity (ALP), hypoxia induced factor (HIF)-1α, and runt-related transcription factor 2 (Runx2) studies signified that the pattern gel could be used in bone regeneration application.

## 2. Materials and Methods 

### 2.1. Materials 

Hyaluronic acid (HA) (MW: 575 kDa) was received as gift from Hanmi Pharmaceutical Co. (Pyeongtaek, Korea), 1,4-butanediol diglycidyl ether (BDDGE, MW: 202 Da), α-MEM and sodium butyrate (NaB) were purchased from Sigma-Aldrich (St. Luis, MO, USA). Sodium hydroxide (MW: 40 Da) was purchased from Yakuri Pure Chemical Co. (Kyoto, Japan). Poly(dimethyl siloxane) (PDMS) (184 Sylgard) was purchased from Dow Corning (Auburn, MI, USA). Penicillin-streptomycin was purchased Lonza Korea (Basel, Switzerland). Cell counting kit-8 (CCK-8) solution was bought from Dojindo Laboratories (Kumamoto, Japan). Live & dead viability/cytotoxicity kit for mammalian cells was purchased from Invitogen (Carlsbad, CA, USA). Dimethyloxalylglycine (DMOG) was procured from Cyman Chemical (Ann Arbor, MI, USA). Anti-RUNX2 antibody, anti-HIF-1-alpha antibody, anti-beta actin antibody, anti-osteocalcin antibody and Goat Anti- Mouse IgG H&L (HRP) were bought from Abcam (Cambridge, UK). RIPA buffer, protease inhibitor, and phosphatase inhibitor were purchased from Sigma-Aldrich (USA). 

### 2.2. Fabrication of Sodium Hyaluronate-BDDGE Patterned Gel (HA-BDDGE)

Fabrication of sodium hyaluronate-BDDGE patterned gel (HA-BDDGE) was performed using the method reported in our previous paper [24]. Briefly, sodium hyaluronate (HA, 0.18 g) was homogeneously mixed in 1 mL of 1% NaOH solution (*w*/*v* %) using centrifuge at room temperature with 10,000 rpm speed for 2 h. Then, 72 µL of BDDGE was added and mixed with a spatula. After that, the mixture was transported into a 10 mL syringe, injected on the PDMS mold supported with teflon-glass slide, and kept 24 h for crosslinking and pattern formation. Afterwards, the crosslinked patterned gel was taken out from the PDMS mold and put in 100% ethanol for 24 h to remove the unreacted reagents. Then, the patterned gel was immersed in phosphate buffered solution (PBS) solution for three days by exchanging the PBS solution after every 12 h. The gel was dried in lyophilizer at −75 °C for further characterizations. 

### 2.3. Characterizations

The attenuated total reflectance Fourier transform infrared (ATR-FTIR) spectra of HA, BDDGE, and dried HA-BDDGE patterned gel were recorded using ATR-FTIR spectrometer (Model: Travel IR, Smiths Detection, Edgewood, MD, USA) in the wavelength range of 650–4000 cm^−1^. The ^13^C NMR analyses of HA and dried HA-BDDGE gel were executed in solid state, while BDDGE was carried out in liquid state with 700 MHz nuclear magnetic resonance (NMR) spectrometer (Model: DD2 700, Agilent Technologies-Korea, Santa Clara, CA, USA). The TGA analyses of HA and dried HA-BDDGE gel were carried out using thermogravimetric analyzer (Model: DTG-60, Shimadzu, Kyoto, Japan) under nitrogen atmosphere. The scan rate was 5 °C/min. The surface morphology of HA and dried patterned HA-BDDGE gel were observed by SEM (Model: SEM, TESCAN VEGA3, Tescan, Seoul, Korea). 

### 2.4. Swelling Study

The % swelling of the HA-BDDGE patterned gel was evaluated gravimetrically. In brief, the pre-weighed dried patterned gel (2*r* = 1 cm) was immersed in 100 mL distilled water at room temperature (25 °C) for 6 h. After a regular interval (1 h), the patterned gel was taken out from distilled water and the surface water was blotted off by tissue paper. Then, the patterned gel was reweighed until equilibrium of their weight was achieved. The % swelling was calculated by the Equation (1):

(1) $$\mathrm{Swelling}\;\left(\text{\%}\right)=\frac{\mathrm{Weight}\;\mathrm{of}\;\mathrm{gel}-\mathrm{Initial}\;\mathrm{dried}\;\mathrm{weight}\;\mathrm{of}\;\mathrm{gel}}{\mathrm{Initial}\;\mathrm{dried}\;\mathrm{weight}\;\mathrm{of}\;\mathrm{gel}}\;\times 100$$

### 2.5. DMOG Loading in HA-BDDGE Patterned Gel and In Vitro Release Study

For DMOG loading inside the HA-BDDGE patterned gel, dried gel samples were immersed into 2 mL of 25, 50 and 100 µM DMOG solutions at room temperature for 2 days in a 12 well plate. After absorption of drug solutions, the gels were dried in lyophilizer at −75 °C.

The release study was performed in distilled water (pH: 7). After 1, 3, 6, 12, 24, 72, 120 and 144 h, aliquots were taken out and absorptions were recorded by UV-Vis spectrophotometer (Model: BioMATE 3, Thermo Scientific, Madison, WI, USA).

### 2.6. In Vitro MC3T3 Cell Culture on the Surface of HA-BDDGE Patterned Gel

An osteoblast precursor cell line derived from Mus musculus (mouse) calvaria (MC3T3, Sigma Aldrich Co., St. Louis, MO, USA) was used after 10 passage for in vitro cell study. The HA-BDDGE patterned gel was sterilized by autoclave (AC-02, Jeio Tech, Daejeon, Korea) for 24 h. Then, the MC3T3 cells at the density of 10,000 cells/cm^2^ were cultured on the surfaces of patterned gel with/without drugs for 7 days. The α-MEM media containing both 10% fetal bovine serum (Gibco Life Science, Waltham, MA, USA) and penicillin-streptomycin (100 unit/mL) was added in the 24 well plate and incubated with 5% CO_2_ at 37 °C. 

Cell adhesion and proliferation were evaluated with the CCK-8 after seeding MC3T3 cells on the surface of the hydrogel. The cell number was counted by the CCK-8 assay with a microplate reader (Tecan, Port Melbourne VIC, Australia). In brief, 100 μL CCK-8 solution was mixed with 900 µL of α-MEM medium in a 15 mL tube. Afterwards, the culture media was removed and the mixed CCK-8 solution was put in the 24 well plate and incubated for 2 h with 5% CO_2_ at 37 °C. After 2 h, 100 μL medium was transferred into a 96 well plate and the optical density was measured at the wavelength of 450 nm by the microplate reader.

### 2.7. Live & Dead Assay

In vitro cell viability and adhesions of the HA-BDDGE patterned gel were observed with MC3T3 cells in the 24-well culture plate. Live & dead viability/cytotoxicity kit for mammalian cells was prepared according to the protocol suggested by the vendor (Invitrogen, Carlsbad, CA, USA). The 1.2 μL of 2 mM ethidium homodimer-1 (EthD-1) and 0.3 μL of 4 mM calcein AM were added into 600 μL PBS and used for live and dead assay. The solution was put in the well plate and incubated for 30 min with 5% CO_2_ at 37 °C. The images of the MC3T3 cells on the HA-BDDGE patterned gel were captured by a fluorescence microscope (Leica DMLB, Wetzlar, Germany). 

### 2.8. Alkaline Phosphatase (ALP) Activity Assay

ALP activities were determined by measuring the amount of *p*-nitrophenol produced using *p*-nitrophenol phosphate substrate. Cell lysates were mixed with alkaline buffer solution and gently shaken for 10 min. ALP substrate was added at room temperature for 30 min. After that, the reaction was stopped with the addition of 0.05 (N) NaOH, and the absorbance at 405 nm was read and compared with a standard curve prepared with *p*-nitrophenol standard solution.

### 2.9. Western Blot Analysis

The in vitro protein expressions of HIF-1α and Runx2 of MC3T3 cells on the patterned hydrogel with/without drugs by using western blot assay. After loading MC3T3 cells at a density of 10,000 cells/cm^2^, cell culture lasted for seven days by employing medium with/without DMOG, NaB. Tris buffered solution (TBS) washing was performed on the cell cultured samples and then radio-immunoprecipitation assay (RIPA) buffer with protease and phosphatase inhibitors was loaded on each well and patterned gels. Cells were harvested from the surfaces by using cell scraper, transferred to a 1 mL microtube in cold and stored at 4 °C in a refrigerator for 30 min. Centrifuge was performed at by using 16,000 rpm at 4 °C for 20 min and then surface layer was transferred to a new microtube. Cell lysate was obtained by heating the cell solutions with Lammeli sample buffer at 95 °C for 5 min, and then transferred into PVDF membrane after loading into DS-PAGE gel. The PVDF membrane was blocked with 5% skim milk solution. Primary antibodies were grafted by incubating with anti-HIF-1α antibody, anti-RUNX-2 antibody, anti-osteocalcin antibody, and then secondary antibodies were done by incubating with goat anti-mouse IgG connected with horse radish protein (HRP). After loading ECL solution in the PVDF membrane with secondary antibody, excitation of drugs was measured with X-ray film by using β-actin as a loading control.

### 2.10. Statistical Analysis

Data were expressed as mean ± standard deviation. Statistical significance was assessed with one-way and multi-way ANOVA by employing the SPSS 18.0 program (ver. 18.0, SPSS Inc., Chicago, IL, USA). The comparisons between two groups were carried out using a *t*-test. The samples were considered as significantly different when *p* < 0.05.

## 3. Results and Discussion 

### 3.1. Fabrication of Sodium Hyaluronate-BDDGE Patterned Gel (HA-BDDGE)

**Scheme 1:**
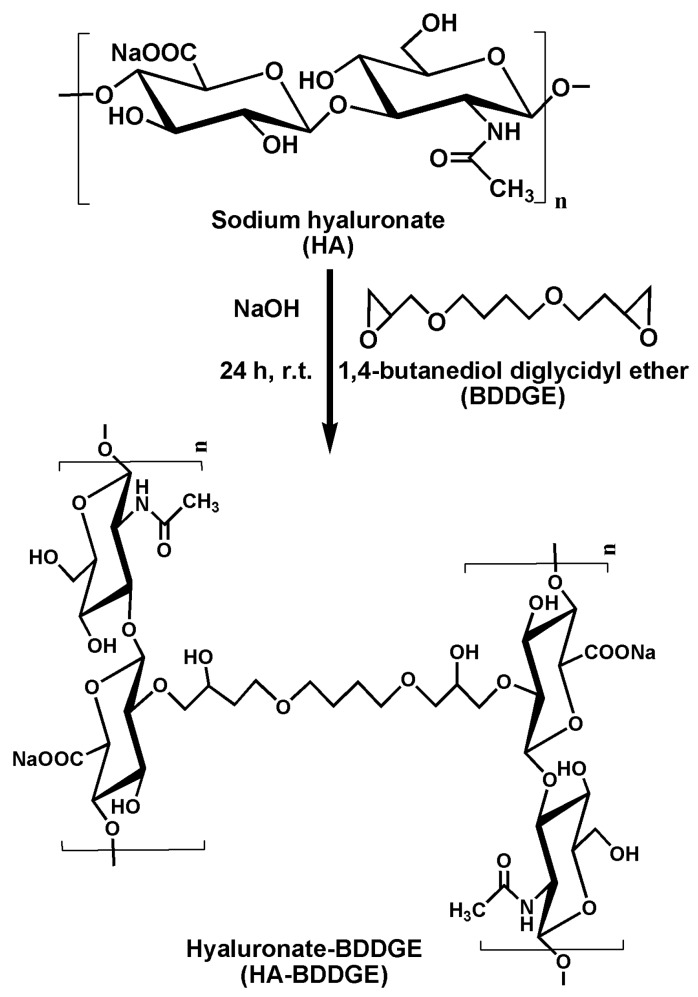
Schematic representation of the reaction between sodium hyaluronate (HA) and 1,4-butanediol diglycidyl ether (BDDGE).

The HA-BDDGE gel was synthesized using HA as biopolymer, BDDGE as crosslinking agent, and NaOH as base. It is assumed that the base (NaOH) abstracts hydroxyl proton from HA and form negative charge over oxygen atom, which act as neucleophilile in the reaction media. The nucleophile attacks on the less hindered electrophilic center of the epoxide rings of BDDGE and opens the epoxide rings. Thus, two HA moieties react with two epoxide rings of BDDGE and form a covalent bond between HA and BDDGE (Scheme 1). Hence, it is supposed that one BDDGE molecule covalently crosslinked two HA molecules through the nucleophilic addition reaction. For the formation of pattern gel, the micro-molding technique was used, where PDMS mold acted as fabrication chamber. The pattern formation in the gel was confirmed by SEM analysis, which is described in the characterization section. 

### 3.2. Characterizations

**Figure 1:**
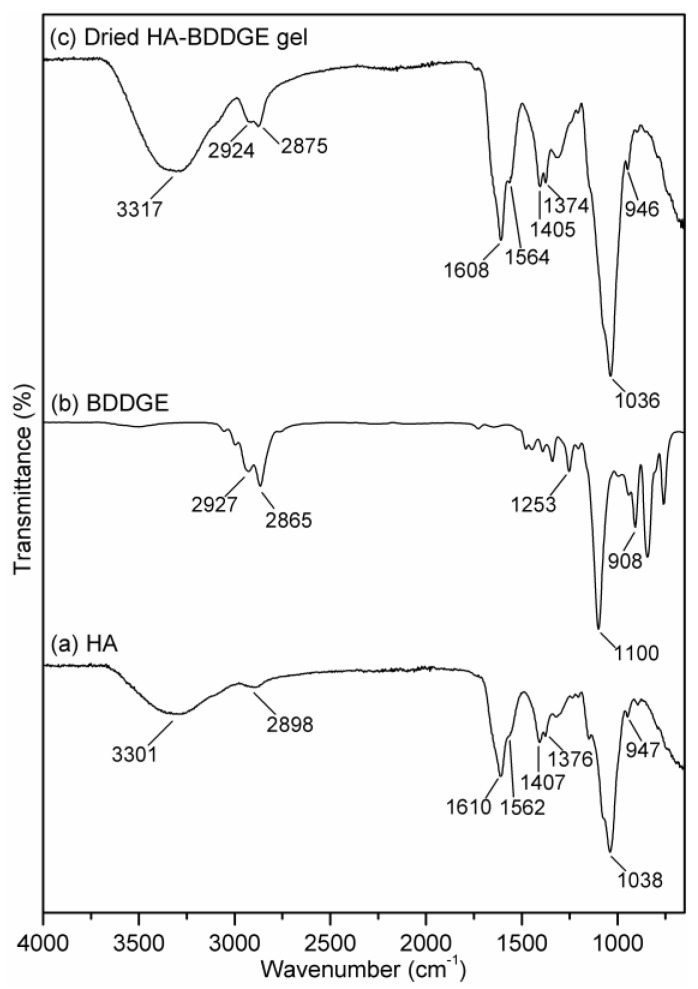
ATR-Fourier transform infrared spectroscopy (FTIR) spectra of (**a**) HA, (**b**) BDDGE, and (**c**) dried sodium hyaluronate-BDDGE patterned gel (HA-BDDGE) gel.

Figure 1 represents the ATR-FTIR spectra of HA, BDDGE, and dried HA-BDDGE gel. In the FTIR spectrum of sodium hyaluronate (HA, Figure 1a), the peaks at 3301, 2898, 1610, 1592, 1407, and 1038 cm^−1^ are because of the stretching vibrations of O–H/N–H bond, C–H bond, C=O bond, amide-II, C–O bond of –COONa group, and C–O–C bond, respectively [41]. The peaks at 1376 and 947 cm^−1^ are due to the vibrations of C–H bending and C–O–H deformation, respectively [41]. In the FTIR spectrum of BDDGE (Figure 1b), the characteristics peaks at 2927, 2865, 1253, 1100, and 908 cm^−1^ are responsible for C–H stretching of epoxy ring, C–H stretching –CH_2_ bond, C–C bond, C–O–C stretching, and C–O stretching vibrations of epoxy ring, respectively [42]. While, in the FTIR spectrum of HA-BDDGE gel (Figure 1c), the peaks at 3317, 2924, 1608, 1562, 1405, 1374, and 946 cm^−1^ are because of the stretching vibrations of O–H/N–H bond, C–H bond, C=O bond, amide-II, C–O bond of –COONa group, vibration C–H bending, and C–O–H deformation, respectively. These peaks suggest the presence of HA moiety in the HA-BDDGE gel. Again, the peaks for C–O–C stretching vibrations of HA and BDDGE moieties merged and gave a peak in the spectrum of HA-BDDGE gel with high intensity at 1036 cm^−1^ (Figure 1c). Most importantly, the disappearance of the peak for C–O bond (908 cm^−1^) of epoxy ring indicates the reaction between –OH groups of HA and epoxy rings of BDDGE (Figure 1c). Whereas, the increase of peak intensity at 3317 cm^−1^ suggest the appearance of new free –OH groups due to the reaction between HA and BDDGE (Figure 1c and Scheme 1).

**Figure 2:**
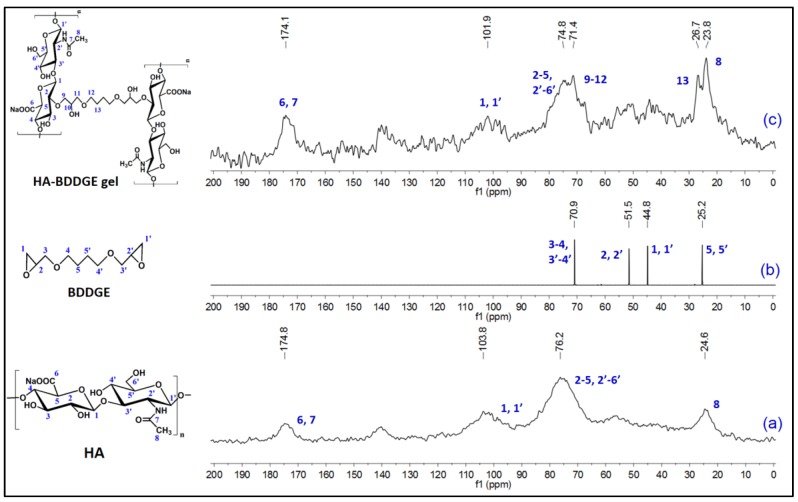
^13^C NMR spectra of (**a**) HA (solid state), (**b**) BDDGE (liquid state), and (**c**) dried HA-BDDGE gel (solid state).

Figure 2 describes the ^13^C-nuclear magnetic resonance (NMR) spectra of HA (solid state), BDDGE (liquid state), and dried HA-BDDGE gel (solid state). In the NMR spectrum of HA (Figure 2a), the chemical shifts at δ = 174.1, 103.8, 76.2, and 24.6 ppm are due to the presence of carbon atoms of C=O groups (C6, C7), anomeric position (C1, C1′), polysaccharide rings (C2–C5, C2′–C6′), and –CH_3_ group (C8), respectively [43]. In the NMR spectrum of BDDGE (Figure 2b), the sharp chemical shifts at δ = 44.8 and 51.5 ppm are owing to the carbon atoms of epoxy rings (C1, C1′) and (C2, C2′), respectively. While, the chemical shifts at δ = 25.2 and 70.9 ppm are because of the chain carbons (C5, C5′) and (C3–4, C3′–4′), respectively (Figure 2b). In the NMR spectrum, HA-BDDGE gel showed the chemical shifts at δ = 174.1, 101.9, 74.8, 71.4, 26.7, and 23.8, which are responsible for the carbon atoms of –C=O (C6, C7), anomeric position (C1, C1′), polysaccharide rings (C2–C5, C2′–C6′), C9–C12 positions, C13, and –CH_3_ group (C8), respectively (Figure 2c). The presence of characteristics peaks for C=O group and anomeric carbon (C1, C1′) and –CH_3_ group (C8) imply the presence of HA in the gel network (Figure 2c). While, the peaks for the carbons of C13 and between C9–C12 confirmed the presence of BDDGE in the gel network and successful formation of HA-BDDGE compound (Figure 2c). 

**Figure 3:**
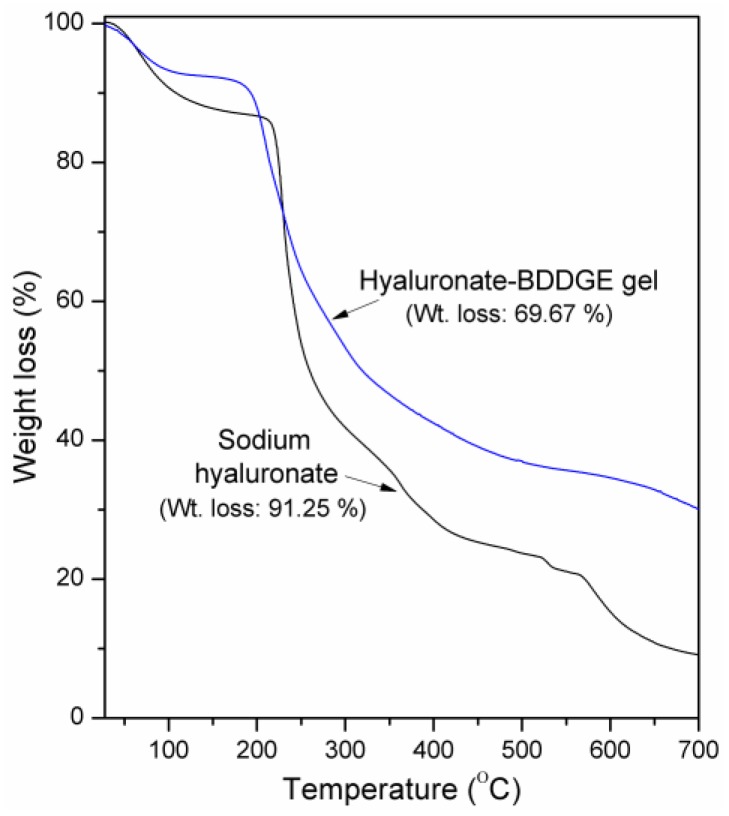
Thermogravimetric analysis (TGA) plots of HA and dried HA-BDDGE patterned gel.

The thermal properties of HA and dried HA-BDDGE gel were analyzed by thermogravimetric analysis (TGA) and results are shown in Figure 3. 

A significant weight loss has been noticed for two samples between the temperature range of 50–520 °C because of the thermal breakdown of HA [44]. The initial weight loss for HA between 28 and 100 °C is owing to moisture evaporation (Figure 3). A sharp weight loss zone is seen between 200–260 °C, then relatively slow decompositions are noticed between 260–400 °C, and 400–700 °C, which are owing to the complete breakdown of polysaccharide residue. The HA showed ~91.25% weight loss in the TGA analysis (Figure 3). In the TGA plot of HA-BDDGE gel, the first weight loss region (28–100 °C) is because of the evaporation of moisture (Figure 3). The steady second (160–310 °C), third (310–500 °C), and fourth (500–700 °C) weight loss zones are due to the decomposition of HA, BDDGE unit and completely breakdown of crosslinked network. The weight loss of HA-BDDGE gel was found as 69.67%, which indicates that the covalent attachment between HA and BDDGE increased the thermal stability of the HA-BDDGE gel.

**Figure 4:**
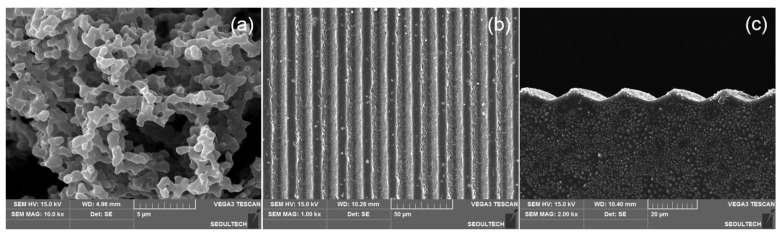
SEM images of (**a**) HA powder, (**b**) surface of HA-BDDGE patterned gel, and (**c**) cross-section of HA-BDDGE patterned gel.

Figure 4 depicts the SEM images of HA and dried HA-BDDGE patterned gel. From Figure 4a, it is observed that HA exhibits an aggregated pod-like structure. After modification with BDDGE, the morphology of HA totally changed and the gel appeared with a relatively smooth surface (Figure 4b). While, the pattern shape is clearly observed from the both surface (Figure 4b) and cross-section images (Figure 4c). It has also been noticed that the width of individual pattern is approximately 5 µm, whereas, the gap between two pattern (valley) is 15 µm (Figure 4b). It is expected that the regular distribution of pattern in the gel structure could be assisted for high adhesion and proliferation of cells.

### 3.3. Swelling Study

**Figure 5:**
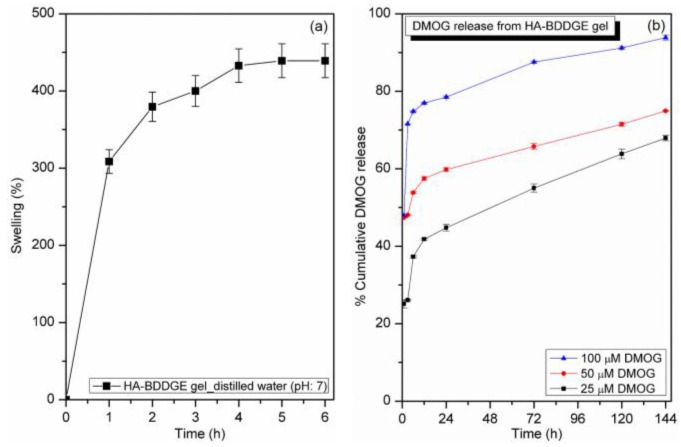
(**a**) Graph of % swelling vs. time for HA-BDDGE patterned gel, and (**b**) in vitro release profile of dimethyloxalylglycine (DMOG) from loaded HA-BDDGE patterned gel over time.

To confirm the gel characteristics of the crosslinked HA-BDDGE polymer, the swelling study was performed with distilled water at 37 °C. Figure 5a represents the swelling study result of HA-BDDGE compound in distilled water at 37 °C.

From the Figure 5a, it is obvious that the rate of water absorption by the HA-BDDGE polymer was higher at the initial stage, then it decreased and finally attained equilibrium swelling state around at 5 h. The HA-BDDGE patterned gel demonstrated the % of swelling as 439 ± 21%. The swelling property confirmed the formation of hydrogel of crosslinked HA-BDDGE polymer in distilled water at 37 °C. 

### 3.4. In Vitro DMOG Release from Loaded HA-BDDGE Patterned Gel

The in vitro DMOG release behavior of DMOG-loaded HA-BDDGE patterned gel with different concentrations of DMOG (25, 50 and 100 µM) is shown in Figure 5b. Release study was performed after the absorption of 25, 50 and 100 µM DMOG. Distilled water (pH 7) was used as medium, while temperature was 37 °C. After 1, 3, 6, 12, 24, 72, 120 and 144 h, absorptions of the aliquots were measured by UV-Vis spectrophotometer (Model: BioMATE 3, Thermo Scientific, Madison, WI, USA). From the release profile (Figure 5b), it is apparent that the initial rate of DMOG release is higher as compared to later stage. This is may be because of the higher rate of swelling of the HA-BDDGE patterned gel and the release of DMOG molecules present on the surface of gel. However, after reaching the equilibrium swelling, the rate of drug diffusion decreased. Among three loaded HA-BDDGE patterned gels (25, 50, and 100 µM), the gel containing 100 µM DMOG exhibited the highest rate of DMOG diffusion (Figure 5b). This is because of the existence of a higher dose of DMOG than that of other grades. In case of 100 µM system, the lesser amount of the % polymer presents in the formulation as compared to 25 and 50 µM systems. Thus, the interaction with HA-BDDGE gel and DMOG will be to certain extent weaker. Besides, the gel layer from which drug molecules diffuse will be weaker and the rate of DMOG release will be faster. After seven days, the % DMOG releases from the HA-BDDGE patterned gel are 93.8 ± 0.6% (for 100 µM), 74.9 ± 0.1% (for 50 µM), and 67.9 ± 0.9% (for 25 µM).

### 3.5. In Vitro MC3T3 Cells Study 

#### 3.5.1. Effect of Drug Molecules of Cell Proliferation

**Figure 6:**
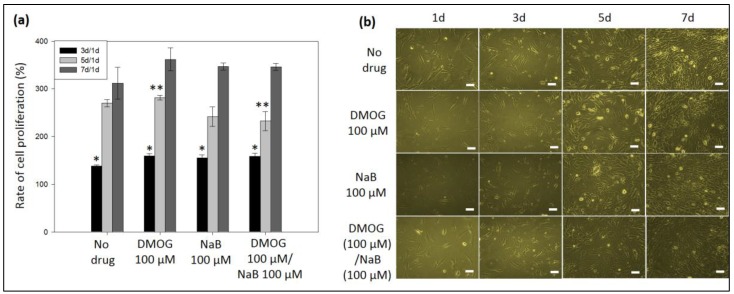
(**a**) Effect of MC3T3 cell proliferation with bolus delivery of drugs (DMOG 100 µM, NaB 100 µM, NaB 100 µM + DMOG 100 NaB 100 µM) on well plate, (**b**) images of MC3T3 cells on well plate by light microscopy (scale bar = 50 µm).

To observe the effects of DMOG and NaB release on cell response, 100 µM of NaB, DMOG and NaB/DMOG were bolus-delivered in 24 well plate after seeding 1 × 10^5^ no. of MC3T3 cells/well with media and the cell culture was lasted for seven days. Cell counting was performed by CCK-8 assay (Figure 6a), while cells images were captured with light microscopy (Figure 6b) at day 1, 3, 5, and 7.

It has been observed that the overall cell proliferation improved when DMOG and NaB were delivered individually or in a combined way (Figure 6a,b). When the proliferation values were normalized with the day 1, the cell proliferation rates were 115 ± 2% and 321 ± 34% at day 3 and 7, respectively (Figure 6a). Importantly, when NaB were bolus-delivered, cell proliferation was improved as 121 ± 2% and 346 ± 33% at day 3 and 7, respectively (Figure 6a). On the other hand, when DMOG were bolus-delivered, cell proliferation was improved as 136 ± 2% and 354 ± 20%, which are similar to those of NaB cases (Figure 6a). In the case of simultaneous delivery of NaB and DMOG, degrees of cell proliferation were 129 ± 2% and 332 ± 14% after day 3 and 7 (Figure 6a). No significant differences in cell proliferations were observed between two drugs.

#### 3.5.2. Effect of DMOG or NaB to MC3T3 Cell Proliferation Cultured on the Surface of HA-BDDGE Patterned Gel 

To observe the effect of DMOG and NaB delivery to MC3T3 cells cultured on the surface of HA-BDDGE patterned gel, cells were culture in native α-MEM medium and the medium with 25 µM, 100 µM, 400 µM DMOG, and NaB drugs. The MC3T3 cells were seeded and *in vitro* cultured on the HA-BDDGE patterned gel at a density of 1 × 10^5^ cells/cm^2^ for seven days. While, cellular behaviors and cell proliferation were observed with light microscopy and CCK-8 assay, respectively. 

**Figure 7:**
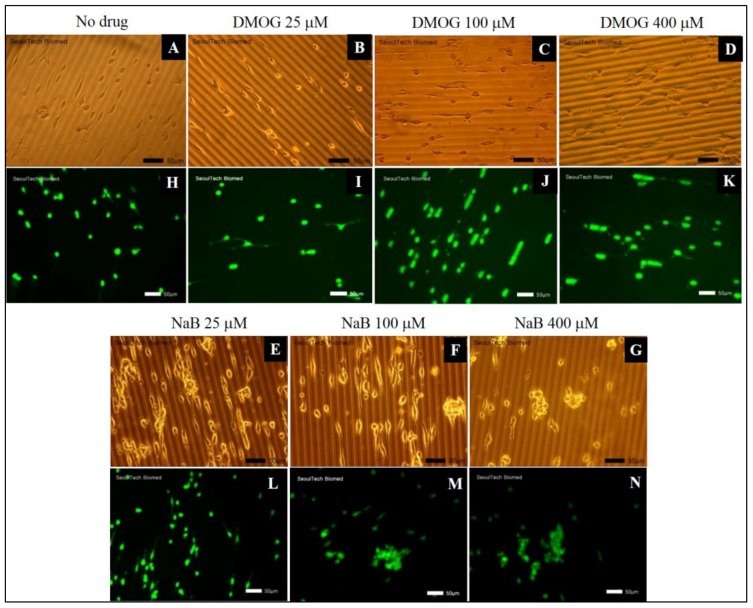
Cellular behavior of MC3T3 cells on the surface of HA-BDDGE patterned gel in absence or presence of DMOG and NaB in medium after 7 days: (**A**,**H**) No drug, (**B**,**I**) DMOG-25 µM, (**C**,**J**) DMOG-100 µM, (**D**,**K**) DMOG-400 µM, (**E**,**L**) NaB-25 µM, (**F**,**M**) NaB-100 µM, (**G**,**N**) NaB-400 µM, (scale bar = 50 µm).

**Figure 8:**
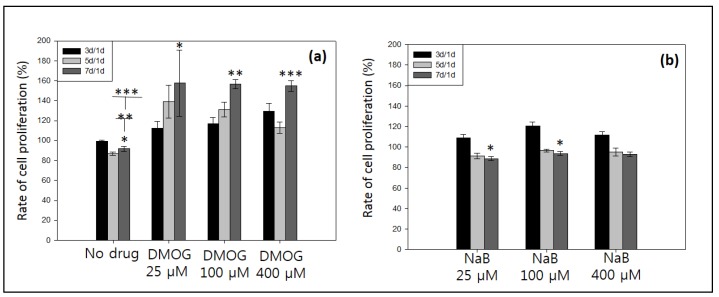
Cell proliferation of MC3T3 cells in absence or presence 25, 100 and 400 µM of (**a**) DMOG, and (**b**) NaB, measured by CCK-8 assay.

According to both LM and L/D assays, it is observed that all of the cells were alive after day 7 (Figure 7A–N). The NaB containing hydrogel showed cells clusters formation. Specifically, the patterned gel with 25 µM NaB showed cell migration on surface (Figure 5L and Figure 7E), whereas, the hydrogel with 100 µM and higher than 100 µM NaB showed cells clusters at day (Figure 7F–N). The patterned gel with DMOG demonstrated better cell migration on surface than NaB (Figure 7B–K). The cell proliferation was measured using CCK-8 assay by normalizing the data of day 3, 5 and 7 by day 1 (Figure 8). The patterned gel with DMOG showed initial quick cell proliferation at day 3, but their proliferations rate decreased when the concentrations of DMOG increased (Figure 8). The patterned gel with NaB showed lower cell proliferation over time (Figure 8).

#### 3.5.3. Effect of DMOG and NaB to MC3T3 Cell Proliferation on the Surface of HA-BDDGE Patterned Gel Depending on the Way of Delivery

**Figure 9:**
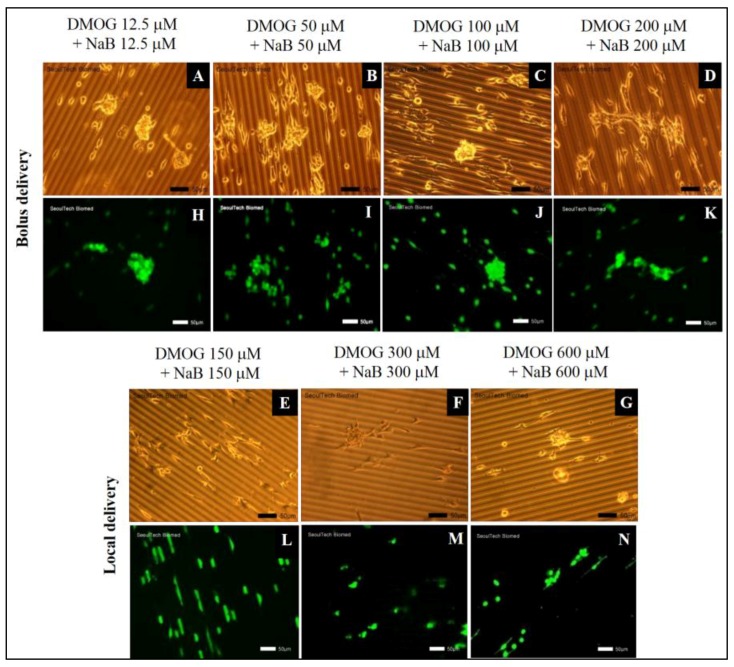
Cellular behavior of MC3T3 cells on the surface of HA-BDDGE patterned gel in presence of both DMOG and NaB after 7 days: bolus delivery (**A**,**H**) NaB/DMOG 12. 5 µM, (**B**,**I**) NaB/DMOG 50 µM, (**C**,**J**) NaB/DMOG 100 µM, (**D**,**K**) NaB/DMOG 200 µM, and local delivery (**E**,**L**) NaB/DMOG 150 µM, (**F**,**M**) NaB/DMOG 300 µM, (**G**,**N**) NaB/DMOG 600 µM.

To detect the cell behaviors on the HA-BDDGE patterned gel, 12.5, 50, 100, 150, 200, 300 and 600 µM of DMOG and NaB were used. The 1 × 10^5^ MC3T3 cells/cm^2^ were employed for in vitro study for seven days. The LM, LD, and CCK-8 tests were performed. For the effects of drugs on cell behaviors drugs were loaded in gel and add in the medium. According to LD assay results at day 7, the medium with both NaB and DMOG induced cell clusters on the patterned HA gel (Figure 9A–D,H–K). For local delivery, the HA gel with either 300 µM or more than 300 µM NaB/DMOG induced cell clusters. But those with 150 µM showed cell high proliferation and cell-cell contact was found on the pattern architecture (Figure 9E–G,L–N). 

**Figure 10:**
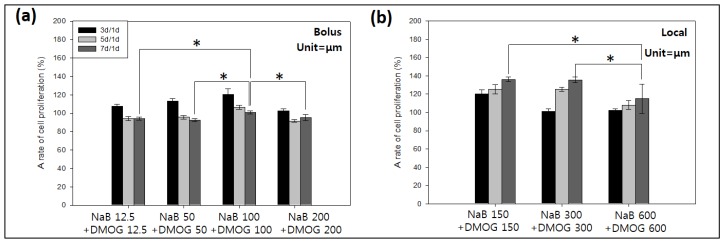
Cell proliferation results of MC3T3 cells in (**a**) bolus, and (**b**) local delivery with 12.5, 50, 100, 150, 200, 300 and 600 µM of DMOG and NaB, measured by CCK-8 assay.

For cell proliferation assays, the system with bolus drug delivery showed the best cell proliferations at the concentrations of 100 µM NaB and DMOG individually (Figure 10a). When the drugs are locally delivered in the patterned gel, cell proliferations lasted longer over time. When both drugs having concentrations of 150 and 300 µM individually, cell proliferations were more effective (Figure 10b and Figure 9E,F,L,M). However, the patterned gel with 600 µM DMOG and NaB low proliferation and induced cell clusters (Figure 10b and Figure 9G,N).

#### 3.5.4. Cell Cluster Formation on the Surface of HA-BDDGE Patterned Gel by Bolus and Local Deliveries of DMOG and NaB

**Figure 11:**
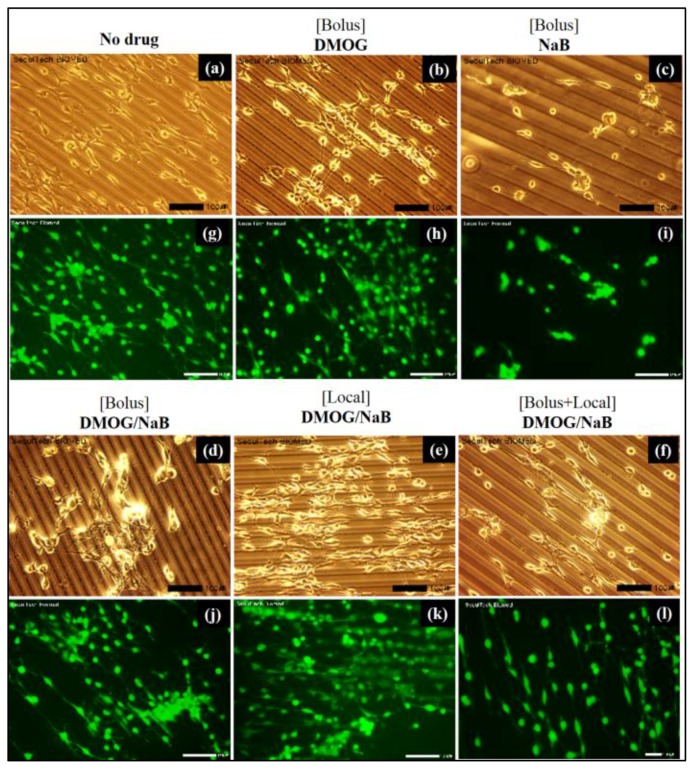
Cell cluster behaviours results: (**a**–**l**) live and dead assay of cells on the HA-BDDGE patterned gel with and without the effects of local and bolus DMOG/NaB delivery.

The cell clusters formation on the patterned HA gel were observed as shown in Figure 11. 

**Figure 12:**
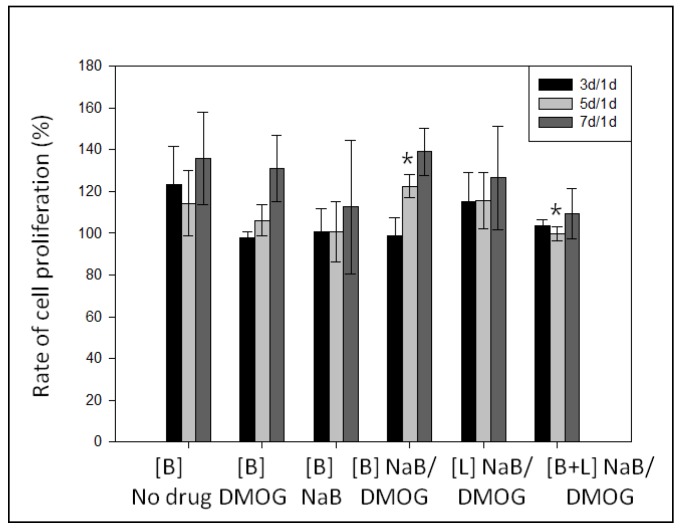
Cell proliferation of MC3T3 cells on the patterned HA gel during cluster formation, measured by CCK-8 assay.

For bolus study, 100 µM DMOG and NaB was added three times (100 µM × 3) during seven days. The same amount of drug loaded gel was used for local delivery. The patterned gel either no drugs or DMOG showed no cell clusters formation (Figure 11). While, the presence of NaB helps the formation of clusters, indicating NaB has vital role for bone regeneration (Figure 11c–l). The NaB either in the medium or inside the patterned gel induced cell cluster (Figure 11c–l). When NaB was introduced in medium, the cell area reduced and the area of clusters increased as the concentration increased. When both NaB and DMOG were added in the medium, surface area of cells decreased significantly. It is also observed that cells were considerably grown on the pattern shape of the gel. The bright field and stain images confirmed that the pattern architecture assisted to form cell-cell contact and association (Figure 11). On the other hand, when DMOG/NaB were added inside the patterned gel, did not showed any significant trends. However, the clusters formation were highest when 300 µM DMOG and NaB were added individually. The presence of NaB, showed lower cell proliferation during cluster formation (Figure 12). 

### 3.6. Effect of DMOG and NaB on Osteogenic or Angiogenic Responses of MC3T3 Cells Cultured on Pattern Gel

**Figure 13:**
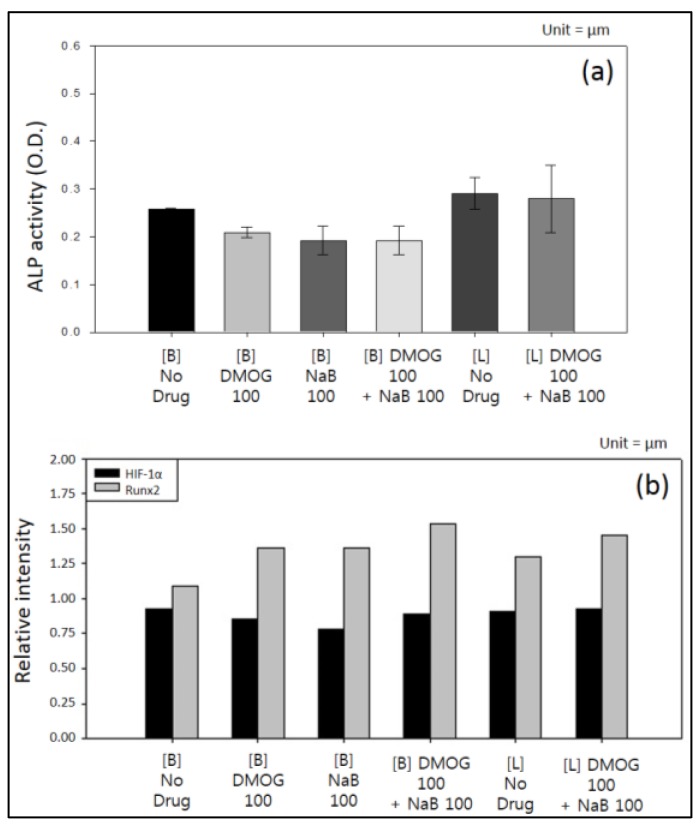
(**a**) Assays of alkaline phosphatase activity, (**b**) and expression of HIF-1α and Runx2 by the delivery of with and without DMOG/NaB through bolus and local modes.

The degree of protein expression was analyzed using MC3T3 cell lines by western blot assay. The results are shown in Figure 13. Combined treatment of 100 µM of both DMOG and NaB through local delivery increased ALP activity (Figure 13a). While, DMOG and NaB individually did not significantly alter the ALP activity. It was well established that NaB increased ALP activity and act as a good marker for the differentiation of MC3T3 osteoblasts [45]. When compared to experiments without drugs, DMOG increased the level of HIF-1α protein (Figure 13b). Combined treatment with DMOG and NaB in both delivery modes also elevated the level of HIF-1α protein. Similar types of results were observed in other reports [46]. Generally, angiogenic activity of cells are interrelated with the HIF-1 pathway [47]. HIF-1 is an oxygen-sensitive compound, which contains HIF-1α and HIF-1β. Under normoxic conditions, HIF-1α is hydroxylated by the enzyme HIF-PH, resulting in ubiquitylation and the degradation of HIF-1α [47]. DMOG is a cell permeable prolyl-4-hydroxylase inhibitor, which can upregulate the protein level of HIF-1α post-transcriptionally under normoxic conditions [47]. In this study, western blotting test showed that the expression level of HIF-1α in MC3T3 enhanced when DMOG was used either individually or through combined treatment with NaB. After MC3T3 cells exposing to DMOG, HIF-1α will be accumulated in cells, and activates HIF-1 complex and shows the expression of various angiogenic genes. 

On the other hand, DMOG, NaB and combined treatments showed high intensities of runt-related transcription factor 2 (Rnx2) expression than that of experiment without drug (Figure 13b). Runx2 is a runt family of transcription factors (Runx1–3) that control the growth and differentiation of various cell lineages [48]. Runx2 protein comprises 128 amino acid Runt domain, which is responsible for the DNA binding. The Runx2 can interact with several proteins including co-regulatory proteins and chromatin remodeling factors, leading to complex role in regulating bone specific genes and differentiation. Mouse MC3T3 cell lines represent Runx2 positive pre-osteoblasts committed to osteogenic lineage [48]. However, for clinical implementation, treatment with rh-BMP2 is essential. As it is reported that the addition of rh-BMP2 to the sites of distraction osteogenesis improves soft tissue healing, and reduced graft exposure and protecting the bone tissue healing [49]. The above in vitro results indicate that DMOG and NaB exerted their angiogenic and osteogenic responses in MC3T3 cells, respectively. Hence, the HA-BDDGE gel could be used in bone regeneration application. 

## 4. Conclusions

The HA-BDDGE gel has been successfully fabricated through nucleophilic addition reaction using NaOH as base. The chemical analyses such as ATR-FTIR, ^13^C NMR, and TGA analyses implied the formation of crosslinked networks. SEM analysis confirmed the pattern architecture on the surface of the gel. The width of pattern was approximately 5 µm, while the width of valley between two patterns was 15 µm. The crosslinked polymer attained equilibrium swelling state after ~5 h in distilled water at 37 °C, which confirmed the hydrogel nature of HA-BDDGE. In vitro release study from DMOG loaded HA-BDDGE patterned gel showed a controlled release nature. The hydrogel is biocompatible against MC3T3 cell lines. In cell proliferation study, the bolus delivery results indicated that the DMOG containing patterned gel demonstrates better cell migration ability on surface than NaB. For local delivery, the pattern gel with 300 µM NaB or 300 µM DMOG induced cell clusters formation, while, the gel with 150 µM NaB/DMOG showed high cell proliferation capability only. The vital role of NaB in bone regeneration has been endorsed from the formation of cell clusters in presence of NaB. The bright field and stain images confirmed that the pattern architecture assisted to form cell-cell contact and association. Through local delivery mode of DMOG and NaB, the HA-BDDGE patterned gel showed higher intensity of ALP, HIF-1α, and Runx2 activities, signify angiogenic and osteogenic responses in MC3T3 cells. However, for clinical application, in vivo study also needs to be explored in the future by modifying some characteristics of gel like porosity, cell adhesiveness, tissue regeneration, and mineralization with inorganic particles or ceramics to improved mechanical properties. Finally, the HA-BDDGE gel, with well micro-patterned architecture, biocompatibility, controlled release ability of DMOG drug, clusters formation ability of MC3T3 cells, and higher intensity in ALP, HIF-1α, and Runx2 studies implied that it could be used in bone regeneration application.
